# Neurocritical Care Society Guidelines Update: Lessons from a Decade of GRADE Guidelines

**DOI:** 10.1007/s12028-021-01375-1

**Published:** 2021-11-02

**Authors:** Lori K. Madden, Venkatakrishna Rajajee, Theresa Human, Mark S. Wainwright, Mary Guanci, Shraddha Mainali, Shaun Rowe, Diane McLaughlin, John Lunde, Abhijit Lele, Herb Fried

**Affiliations:** 1 Center for Nursing Science, University of California Davis Health, 2315 Stockton Blvd, North Addition 3015, Sacramento, CA 95817 USA; 2 Departments of Neurosurgery and Neurology, University of Michigan, Ann Arbor, MI USA; 3 Cumberland Pharmaceuticals, Nashville, TN USA; 4 Division of Pediatric Neurology, University of Washington, Seattle, WA USA; 5 Neuroscience Intensive Care Unit, Massachusetts General Hospital, Boston, MA USA; 6 Department of Neurology, Virginia Commonwealth University, Richmond, VA USA; 7 Department of Clinical Pharmacy and Translational Science, College of Pharmacy, The University of Tennessee Health Science Center, Knoxville, TN USA; 8 Departments of Neurology and Neurocritical Care, University of Florida Health, Jacksonville, FL USA; 9 Division of Critical Care, Intensive Care Consortium/Orange Park Medical Center, Orange Park, FL USA; 10 Departments of Neurosurgery and Anesthesiology and Pain Medicine, University of Washington, Seattle, WA USA; 11 Division of Neurosurgery, Denver Health Medical Center, Denver, CO USA

**Keywords:** Evidence-based medicine, Grading of recommendations assessment, Development and evaluation, Clinical practice guideline, Neurocritical care, Methodology, Systematic review

## Introduction

The Neurocritical Care Society (NCS) launched its clinical practice guideline program in 2012 with the publication of “Guidelines for the Evaluation and Management of Status Epilepticus” [1]. Work on this guideline coincided with the development of standards for trustworthy clinical practice guidelines by the Institute of Medicine (now the National Academy of Medicine) in 2011 [2]. These standards highlighted the need for rigorous and consistent evidence-based methodology aimed at producing clear, actionable recommendations relevant to the individual patient encounter. The NCS Guideline Committee (GC) made the prescient decision to adopt and implement an emerging methodology, Grading of Recommendations Assessment, Development and Evaluation (GRADE) [3], which has since been adopted or endorsed by more than 100 organizations worldwide. In the past decade, the US GRADE Working Group has elaborated, refined, and extended this methodology in many publications.

With the emergence of multiple GRADE-based guidelines since standards were developed, concerns were also raised about lack of adherence to published GRADE criteria in the development of these guidelines. In 2020, the US GRADE Working Group critically reviewed GRADE guidelines published in the National Guideline Clearinghouse [4]. Of the guidelines studied in this sample, which included two from NCS, nearly half did not comply with one or more of the key GRADE criteria for appropriate application of the methodology. Most commonly lacking were GRADE evidence profiles and evidence summaries, as well as explicit consideration of all four central domains required to move from evidence to recommendation. Those domains include certainty of evidence, balance of benefits to harms, patients’ values and preferences, and resource use and equity.

Over the course of the past decade, NCS has produced a number of guidelines and related projects, the majority of which used methodology derived from GRADE publications (see Table 1). However, as our understanding of the requirements of GRADE deepened, it became clear that our application of the methodology across projects was inconsistent and generally fell short of the necessary rigor in various ways. On the basis of this growing insight, the NCS GC developed and is implementing a number of significant improvements in our guideline procedures.

**Table 1:** NCS guidelines and projects to date

Title	Year	Type	GRADE
Critical Care Management of Patients Following Aneurysmal Subarachnoid Hemorrhage	2011	Consensus statement	+
Guidelines for the Evaluation and Management of Status Epilepticus	2012	Guideline	+
Consensus Summary Statement of the International Multidisciplinary Consensus Conference on Multimodality Monitoring in Neurocritical Care	2014	Consensus statement	+
Evidence-Based Guidelines for the Management of Large Hemispheric Infarction	2015	Guideline	+
Guideline for Reversal of Antithrombotics in Intracranial Hemorrhage	2015	Guideline	+
Prophylaxis of Venous Thrombosis in Neurocritical Care Patients	2015	Guideline	+
Recommendations for the Critical Care Management of Devastating Brain Injury: Prognostication, Psychosocial, and Ethical Management	2015	Position statement	+
The Insertion and Management of External Ventricular Drains: An Evidence-based Consensus Statement	2016	Consensus statement	+
The Implementation of Targeted Temperature Management: An Evidence-Based Guideline from the Neurocritical Care Society	2017	Guideline	+
Standards for Neurologic Critical Care Units	2018	White paper	−
Clinical Performance Measures for Neurocritical Care	2019	White paper	−
Guidelines for the Acute Treatment of Cerebral Edema in Neurocritical Care Patients	2020	Guideline	+
Neurocritical Care Resource Utilization in Pandemics	2020	Position statement	−
Critical Care Management of Patients Following Aneurysmal Subarachnoid Hemorrhage Update	Est 2021	Guideline	+
Antiepileptic Drug Prophylaxis in Neurocritical Care	Est 2022	Guideline	+
Neuroprognostication in Neurocritical Care	Est 2022	Guideline	+
Guidelines for the Evaluation and Management of Status Epilepticus Update	Est 2023	Guideline	+

Est, Estimated completion date; GRADE, Grading of Recommendations Assessment, Development and Evaluation; NCS, Neurocritical Care Society; + , GRADE methodology consulted for product development; − , No GRADE methodology utilized

The aim of this article is to introduce and explain the basis for these changes. To that end, rather than reviewing in detail individual NCS projects with widely varying formats and objectives and rotating leadership, we chose to begin by setting forth the requirements for consistent and trustworthy GRADE guidelines. On the basis of the lessons learned from an examination of our own published projects and those of other societies, we summarize the changes necessary to bring our guidelines up to the highest contemporary standards.

Thus, between December 2019 and June 2020, the NCS GC undertook a project to review existing best practices for guideline development using a comprehensive survey. For this purpose, we selected organizations with specialty interests overlapping with the NCS and others that have adopted GRADE methodology for guideline development (see Table 2 for full list). The GC members examined published policies and procedures for each organization and, when possible, contacted a member of the organization’s guidelines committee for additional questions and clarifications (see Supplemental Content Table 1). Note that details of the identified organizations’ procedures are based on the authors’ best understanding using available information during this limited time window and may have changed since then. Through the review process, we have identified three broad themes in guideline development: (1) topic, scope, and panel; (2) systematic review; and (3) developing recommendations. Below, we discuss NCS methodology for guideline development under these three major themes.

**Table 2:** Organizations identified for GC survey

Neurocritical care society
Agency for Healthcare Research and Quality
American Academy Neurology^a^
American College of Chest Physicians
American Academy of Family Physicians
American Academy of Nurse Practitioners
American Association of Critical Care Nurses^a^
American Association of Neurological Surgeons/Congress of Neurological Surgeons^a^
American Association of Neuroscience Nurses^a^
American College of Surgeons, Trauma Quality Improvement Program
American Epilepsy Society^a^
American Heart Association^a^
Brain Trauma Foundation^a^
Centers for Disease Control and Prevention^b^
Eastern Association for the Surgery of Trauma^a,b^
Endocrine Society^b^
European Society of Intensive Care Medicine^a^
European Stroke Organization^a^
Hospital Infection Control Practices Advisory Committee
Latin American Brain Injury Consortium^a^
Society for Neuroscience in Anesthesia and Critical Care^a^
Society of Critical Care Medicine^a,b^
US Preventive Services Task Force^a^
World Health Organization^b^

*GC* guideline committee, *GRADE* grading of recommendations assessment, development and evaluation

^a^Overlapping interest

^b^Known to use GRADE methodology

## GRADE Guideline Methodology

### Topic, Scope, and Panel

Regardless of methodology, the success of a guideline project depends greatly on a systematic approach and planning at inception. Topic selection, determination of scope, and identification of key clinical questions are critical elements in determining the feasibility of the project. Criteria developed by the GC for topic selection and current GRADE guidance for the development of clinical questions are discussed in detail below.

The guideline co-chairs are selected from a pool of subject matter experts from multidisciplinary backgrounds who have demonstrated skill in managing complex projects and ideally have experience in the application of GRADE methodology. After identification of guideline co-chairs, selection of the writing panel is the next priority. Panel members may be selected from a pool of volunteer NCS members or may be nominated by the co-chairs with GC oversight. Although these members will also be subject matter experts, it is not required that they have experience with GRADE. The key principle is that the panel must be diverse and must attempt to represent all the stakeholders affected by the recommendations developed by the guideline. Accordingly, the NCS GC aims for a balance among physicians, nurses, pharmacists, physician assistants, and advanced practice nurses. Type of practice setting (academic versus community) and geographic diversity are considered, along with traditional metrics of diversity among panel members. Panelists from varied related specialty areas, such as rehabilitation, neurological surgery, epileptology, or other relevant areas, may also be included on the basis of the topic. A more recent addition to this process involves engagement with health care consumers. As such, the panel will recruit patient or family representatives. Incorporation of the patient/family perspective can be invaluable in deciding on patient-centered outcomes and offering insight into the impact of recommendations.

It is often difficult for organizations to assemble and retain an internal team of methodologists with adequate expertise in GRADE and experience in performing systematic reviews. Basic criteria for this role have been suggested by the GRADE Working Group. Our survey has shown that many organizations enlist professional methodologists for this purpose (see Table 3). There are important benefits in this approach, which is discussed below.

**Table 3:** Organizations and selected guideline practices

Organization	GRADE methodology Y/N	External methodologist Y/N	Public member Y/N	Good practice statements Y/N
NCS	Y	Y	Y	Y
AACN	N	N	N	Y
AAN	N	N	Y	Y
AANN	Y	N	N	N
AANS/CNS	N	Y	N	N
ACCP	Y	Y	Y	Y
AHA	N	N	N	N
CDC	Y	N	Y	Y
EAST	Y	N	N	Y
Endocrine Society	Y	Y	Y	Y
ESICM	Y	N	N	Y
SCCM	Y	Y	Y	Y
USPSTF	N	Y	Y	N
WHO	Y	Y	Y	N

AACN, American Association of Critical Care Nurses; AAN, American Academy of Neurology; AANN, American Association of Neuroscience Nurses; AANS, American Association of Neurological Surgeons; ACCP, American College of Chest Physicians; AHA, American Heart Association; CDC, Centers for Disease Control and Prevention; CNS, Congress of Neurological Surgeons; EAST, Eastern Association for the Surgery of Trauma; ESICM, European Society of Intensive Care Medicine; N, no; NCS, Neurocritical Care Society; SCCM, Society of Critical Care Medicine; USPSTF, US Preventive Services Task Force; WHO, World Health Organization; Y, yes

### Systematic Review

**Fig. 1:**
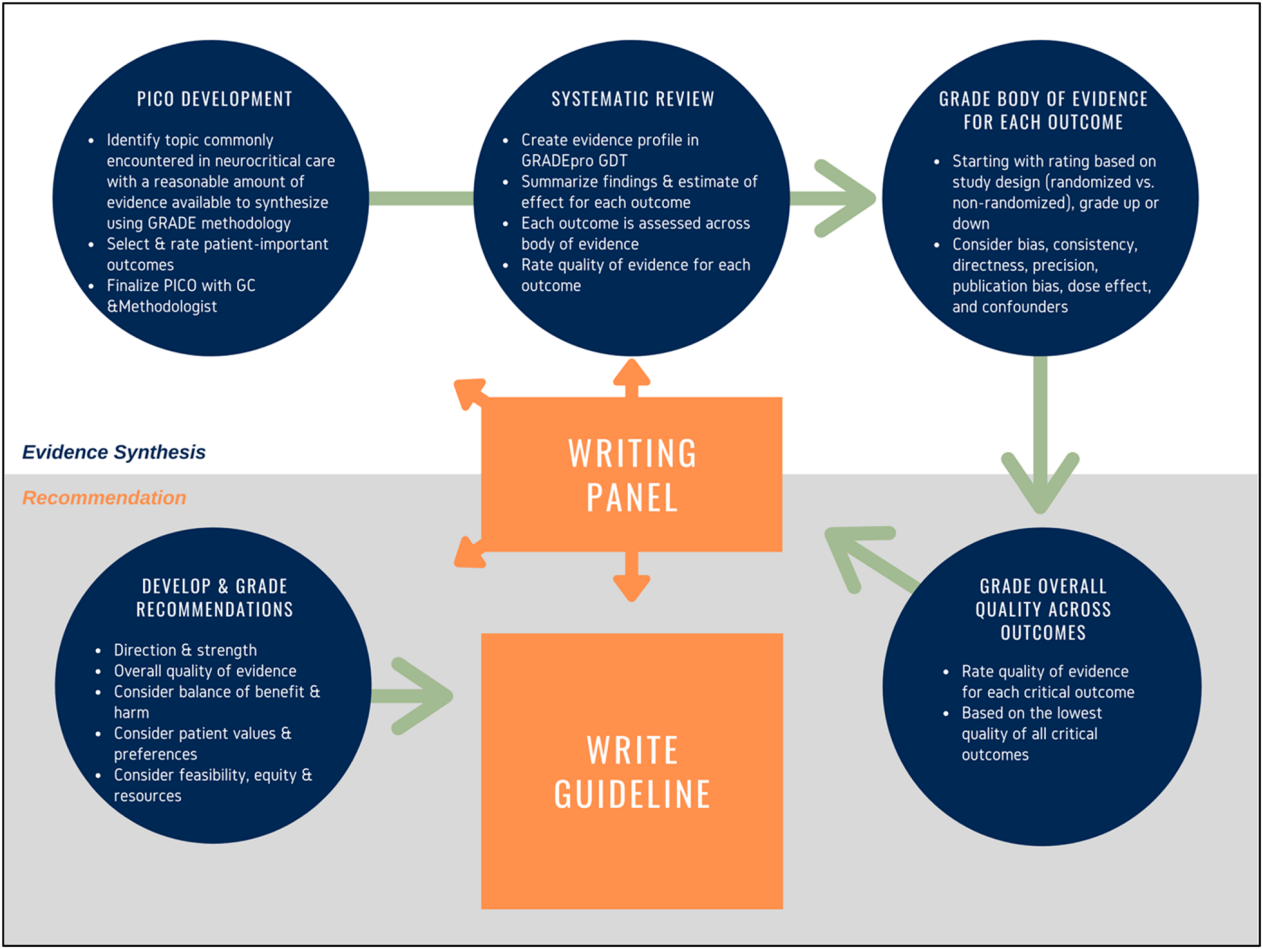
Schematic view of guideline development process. GC Guideline Committee, GRADE Grading of Recommendations Assessment, Development and Evaluation, PICO population, intervention, comparator, and outcomes, GDT guideline development tool. Adapted from Andrews J, Guyatt G, Oxman AD, et al. GRADE guidelines: 14. Going from evidence to recommendations: the significance and presentation of recommendations. J Clin Epidemiol. 2013;66:719–25

GRADE is a comprehensive tool for rating the quality of evidence and grading the strength of recommendations for specific clinical questions (see Fig. 1 for schematic of the GRADE approach). The vehicle for this process of acquiring and assessing or adjudicating the evidence is the systematic review (SR). An SR is a highly structured process that aims to generate reproducible findings and conclusions from a given set of data, that is, were two different panels to follow this rigorous process, they should ideally arrive at the same result from a given evidence base. Consistency and transparency in methods and judgments are required to achieve this.

The first step in the SR is the careful crafting of focused clinical questions [5]. The generally accepted format is PICO: population, intervention (or diagnostic test) of interest, comparator (another treatment, gold standard diagnostic test, or usual care), and outcomes (which must include patient-important benefits as well as harms). Although timing and setting are usually considered to be features of outcome and population, respectively, they may be added (PICOTS) when the panel wishes to highlight them. Finally, in our experience from a practical standpoint, a range of six to fifteen PICO/PICOTS questions is preferred.

The PICO question unifies all the steps in the SR and recommendation process. Each PICO element must be clear and specific or the entire SR project may be compromised. For example, the population may include heterogeneous subgroups with different baseline risks for an outcome. Multiple interventions may require a more sophisticated network analysis. Lack of a clear comparator may make it difficult or impossible to generate an effect size for the intervention. If not strongly linked to patient-important outcomes, use of surrogate outcome measures (such as biomarkers) may require downgrading of the evidence quality.

Once PICO questions have been refined with up to seven patient-important outcomes that have been identified with ranking of relative importance (critical, important, and not important), a search strategy is developed with a research librarian. Important trade-offs between global inclusiveness (e.g., whether to consider multiple language sources, unpublished or “gray” literature, etc.) and feasibility (retrieving a large number of irrelevant or unusable studies) require close collaboration between the panel and the information specialist. Search strategies that are too broad will produce an avalanche of irrelevant studies, whereas stringent strategies are likely to miss important evidence. Once a search strategy is applied, the articles are then screened for inclusion or exclusion by using SR software (e.g., DistillerSR or Covidence). The vast majority of references are removed via screening in this multilayered, labor-intensive process.

The next task, data extraction from the selected studies, introduces important innovations of the GRADE approach. First, it does not assign an evidence quality rating (high, moderate, low, or very low) to any individual study. Rather, evidence quality (certainty in the evidence) is an attribute of the entire body of evidence for a given outcome. What GRADE evaluates in each study is its risk of bias (otherwise known as study limitations). In this process, randomized controlled trials (RCTs) are initially considered to be of high quality because randomization is felt to be the only secure way to eliminate residual confounding and bias in patient selection; nonrandomized (observational) studies (NRS) begin as low quality.

In neurocritical care, there is a paucity of high-quality evidence, particularly in terms of well-designed and well-executed RCTs. Thus, there is increasing interest in the wide variety of NRS. Cochrane’s risk of bias tool uses domains familiar to many as the appropriate instrument to apply to RCTs [6]. Many tools have been proposed and used for NRS (Newcastle–Ottawa Scale, for example) [7]. However, a new and more detailed tool, the Risk of Bias In Nonrandomized Studies of Interventions (ROBINS-I) has been introduced in GRADE to evaluate NRS [8, 9], and other tools are available for diagnostic tests (QUality Assessment of Diagnostic Accuracy Studies-2) or for prognostic studies (Prediction model of Risk Of Bias ASessment Tool and QUality In Prognostic Studies, for example) [10, 11].

The second important innovation of GRADE is that evidence is aggregated, and quality of evidence is assessed, according to the critical and important outcomes prespecified in the PICO question [12]. That is, GRADE is “outcome centric” and recognizes that the quality of evidence available for each outcome may often be different.

**Fig. 2:**
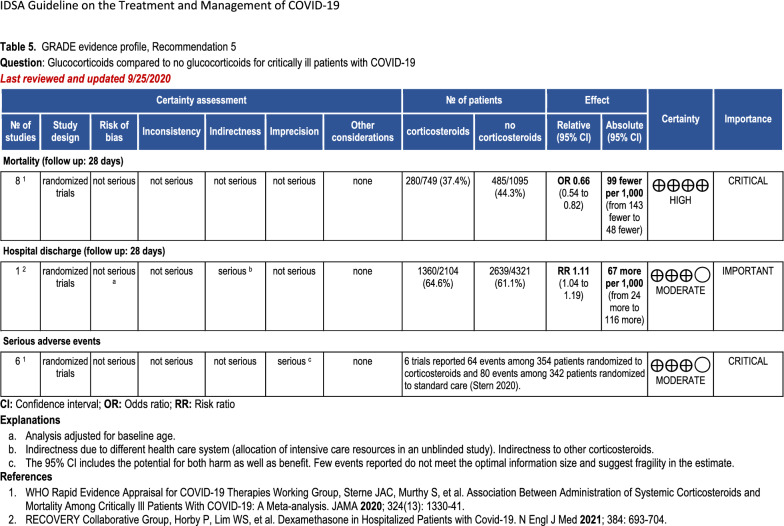
Example of GRADE evidence profile. GRADE Grading of Recommendations Assessment, Development and Evaluation, IDSA Infectious Diseases Society of America. Table courtesy of the Infectious Diseases Society of America. Original available at https://www.idsociety.org/practice-guideline/covid-19-guideline-treatment-and-management/

Risk of bias is only the first of the domains GRADE uses for assessing the quality of a body of evidence (see Fig. 2). Four other domains may result in downgrading a body of evidence for an outcome: inconsistency between studies, indirectness, imprecision, and other considerations, including publication bias. GRADE considers three domains that might result in rating up: magnitude of effect, dose–response gradient, or plausible unmeasured confounders, which might increase or decrease the effect, if present. The writing panel then makes judgments about whether limitations in each domain are serious or very serious. The quality of evidence for each outcome is then globally adjudicated by the panel. Guideline developers (but not systematic reviewers) then review all the information to make a final decision about which outcomes are critical and which are important. This concludes with a final decision regarding the rating of overall quality of evidence across critical outcomes for the clinical question.

An existing high-quality published SR could be adapted or adopted for use in a new guideline, but more commonly, a new SR is performed by the writing group itself. This has been the practice for NCS guidelines. Our experience over a decade of work has highlighted two interrelated problems with this approach. The first problem is that to apply GRADE methodology properly, dedicated study, training, and experience are required. This is difficult to achieve in a volunteer organization, such as NCS, in which there is little carryover in writing group personnel from one project to another, resulting in a lack of consistency and institutional memory.

A second problem, which contributes to the first, is the heavy workload encountered by the panel of volunteer subject matter experts in screening and extracting data from hundreds or thousands of studies. Knowledge and experience gained in each project do not carry forward seamlessly to the next. Experienced panelists find it difficult to commit to another project, realizing the labor required to complete a guideline. Moreover, in recent years there have been many important advances and refinements in GRADE methodology and in clinical epidemiology; the earliest GRADE publications lacked necessary specific and detailed guidance. Thus, a guideline methodologist faces the additional challenge of providing expertise in a field undergoing rapid evolution.

With the widespread adoption or endorsement of GRADE by medical societies and health care organizations worldwide, the need for appropriately trained methodologists to assist in creation of high-quality GRADE guidelines has become paramount. Inappropriate use of GRADE methodology has been described by senior GRADE authors [4]. The GRADE Working Group has suggested a set of fundamental proficiencies and experiences that would be desirable in a methodologist, which include (1) basic knowledge of statistics, study design, and epidemiology; (2) GRADE-specific training through workshops, webinars, familiarity with all articles in the *Journal of Clinical Epidemiology* in the GRADE series, and the GRADE handbook; (3) experience with producing at least three GRADE/Cochrane SRs, with meta-analyses, and GRADE evidence profile/summary of findings tables using GRADEpro Guideline Development Tool software; (4) familiarity with using the ROBINS-I tool to evaluate NRS; and (5) experience using the Evidence to Decision Frameworks to arrive at recommendations.

It is not necessary for the methodologist performing an SR to be a subject matter expert. In fact, in a voluntary society, such as NCS, it may be particularly challenging to find volunteers with appropriate background training in GRADE who would also be available to commit their time to a series of guideline projects. On the basis of our experience and the survey result, there is an increasing trend for medical societies and health care organizations to rely on outside experts given the above-mentioned challenges.

### Developing Recommendations

A defining feature of GRADE is the structured manner in which it moves from evidence to recommendation. An SR produces an evidence summary and judgment of certainty in the evidence that is relevant for the specified population. What is lacking in an SR, and what is required in a guideline, is a framework for describing the context of the individual patient encounter.

Each PICO question should be answered by a clear, actionable recommendation, not a statement of fact. GRADE describes recommendations in terms of strength (strong or conditional/weak) and direction (for or against a management strategy). Strength refers to “the extent to which we can be confident that the composite desirable effects of a management strategy outweigh the composite undesirable effects” [13]. It is for this reason that GRADE provides a separate rating of evidence quality for each outcome. GRADE evidence profiles and summary of findings tables are organized accordingly.

However, the balance of benefit and harm is not necessarily the same for each clinical circumstance, and concepts such as “applicability” used in some guidelines are too vague and undefined to provide useful guidance [4]. Thus, GRADE has created Evidence to Decision Frameworks, which specify four essential domains to be explicitly considered [14, 15]:

- Certainty in evidence of effect of the management strategy
- Values and preferences of the patient/caregiver
- Balance of desirable and undesirable effects, from the patient’s perspective
- Resource implications, acceptability, and equity

A strong recommendation implies that “all or almost all informed people would make the recommended choice for or against an intervention” [16]. A conditional (weak) recommendation implies that “most informed people would choose the recommended course of action, but a substantial number would not” [16]. Thus, a strong recommendation suggests uniformity; a conditional one suggests variability. Such conditional recommendations should be accompanied by an explicit statement of the factors that might influence the decision.

Great emphasis is placed on determining the patient’s perspective and circumstances as well as the relative importance of various outcomes to that individual. Together with the best available evidence and clinician expertise, explicit consideration of the individual patient’s circumstance is what defines evidence-based practice [17]. How can this be achieved? Ideally, one would use empirical evidence from an SR that considers the importance that patients perceive about various health outcomes or utilities. GRADE has provided detailed guidance on how to evaluate such data [18, 19]. However, studies that included patients’ perceptions of outcome importance are seldom available for neurocritical care. Panels consisting of subject matter experts will base their estimate of patients’ values and preferences on their own clinical experience, with all the forms of bias that accompany such estimates. It is for these reasons that, as our survey has demonstrated, guideline panels increasingly incorporate public representation, commonly as patients and families/caregivers, to enrich their own perspective. This can be particularly helpful at the early stage of identifying and ranking outcomes in the generation of PICO questions, as well as in considering the impact of final recommendations.

A common and vexing problem arises when the evidence base for a PICO question proves to be of low or very low quality. Low (“the true effect may be substantially different from the estimate of the effect”) or very low certainty (“the true effect is likely to be substantially different from the estimate of the effect”) are encountered frequently in neurocritical care because many of the clinical studies available are NRS. GRADE assigns an initial “low” quality rating to all such studies, and deficiencies in design and conduct may lead to rating down the body of evidence further to “very low.” In this circumstance, guideline panels may be tempted to create recommendations that are not explicitly supported by an analysis of evidence quality. These may be termed “good practice” or “best practice” statements. These statements essentially represent the expert opinion (which GRADE does not consider to be evidence) of the panel. GRADE provides an analysis of when these may be justified [20] and suggests asking the following questions:

1. Is the statement clear and actionable?
2. Is the message really necessary?
3. Is the net benefit large and unequivocal?
4. Is the evidence difficult to collect and summarize?
5. Has the rationale been made explicit?
6. Should this statement be GRADE-ed? Does a large body of indirect evidence that can be convincingly linked to the PICO support the management strategy?

Thus, although there may be instances when these questions can be answered affirmatively, we recognize that overuse of such statements carries the risk of diminishing the confidence of guideline users in the rigor and trustworthiness of the guideline. The US Preventive Services Task Force specifically advises that “decision makers do not have the luxury of waiting for certain evidence. Even though evidence is insufficient, the clinician must still provide advice, patients must make choices, and policy makers must establish policies” [21]. This is consistent with GRADE guidance, which specifies the following:

Clinicians will rarely explore the evidence as thoroughly as a guideline panel, nor devote as much thought to the trade-offs, or the possible underlying values and preferences in the population. We therefore encourage panels to deal with their discomfort and to make recommendations even when confidence in effect estimate is low and/or desirable and undesirable consequences are closely balanced. Such recommendations will inevitably be weak, and may be accompanied by qualifications [16].

### Summary of Lessons Learned

Review of a decade of work by NCS guideline panels together with a survey of the guideline practices of other organizations has revealed a number of opportunities for improvement in the conduct and methodological rigor of our clinical practice guidelines.

### Common Themes from Successful Guideline Programs

The crucial role of the methodologist was evident in the GC survey. Thus far, NCS has used members who have undergone GRADE workshop training as methodologists. Other surveyed organizations employ staff who serve as methodologists across projects. Such individuals have deep experience and strong institutional support from other internal experts. These organizations also have a long history of creating guidelines that closely follow a well-established methodology, whether GRADE or other. Institutional memory is key.

The most successful guideline programs in our survey use professional methodologists to perform the SR and to produce the evidence synthesis, with evidence tables and meta-analysis meeting GRADE or Cochrane standards. Close collaboration between the methodologist, the panel chairs, and the oversight body (GC or other) throughout the project was a common theme. This coordination ensures that the topic and scope are both appropriate and feasible and that PICO questions are correctly formulated. The methodologist provides the writing panel with education and mentoring regarding the GRADE process so that additional advanced training in GRADE for chairs and panelists may not be needed. In addition, the methodologist benefits from frequent clinical guidance as the SR progresses.

### Key Challenges with Previous NCS Practices

NCS has not developed a sufficient pool of experienced members with the ability to work intensively on successive projects. The GC has found that attendance at one or more workshops does not make one a methodologist. Competence in these techniques cannot be gained from slide presentations or by reading summary reviews. Past guideline chairs have typically not had formal training in GRADE or SR methodology. They have relied on an NCS member volunteer who has served as a GRADE methodology advisor after one or more guideline experiences and attendance at a GRADE workshop.

Panel members have usually been overwhelmed by the large commitment of time and effort involved in a formal SR. Inexperience with GRADE methodology may add to the burden on chairs as well as panelists. This may result in evidence summaries that deviate significantly from GRADE principles and practice, incorrectly formulated PICO questions, chosen topics with a scope that is excessive in relation to what is feasible, and recommendations in which the linkage to evidence quality is inappropriate and the rationale is unclear. Importantly, inexperience has contributed to significant difficulty in conforming to a clear timeline.

### Key Improvements Going Forward

#### Scope and Topic Selection

Suggestions for guideline topics will be solicited from the GC and NCS membership. However, the process of choosing a topic will rely on established criteria and compatibility with GRADE methodology. Adherence to the following criteria can avoid common pitfalls and contribute to the success of future guideline efforts:

- The topic is commonly encountered in neurocritical care
- There is equipoise between different management strategies, resulting in significant variation in practice
- A reasonable amount of evidence is currently available from clinical trials and can be synthesized by using GRADE methodology
- The topic lends itself to 6–15 tightly constructed clinical questions with both defined populations or subgroups and critical and important outcomes that lend themselves to quantitative analysis
- There is sufficient overlap between adult and pediatric care that questions can address both populations, when appropriate and possible
- There has not been a recent high-quality guideline directly addressing the topic
- The topic has not been the subject of current or recent NCS projects

#### Panel Composition

The GC will make deliberate efforts to assemble a diverse panel of stakeholders across health care organizations, genders, roles, and specialties. The GC is also committed to including public members for the perspectives of patients and caregivers.

#### PICO Formulation

Panel chairs will draft PICO questions with input from the writing panel. These draft questions will be reviewed by the GC and by the methodology team prior to forwarding to an information specialist working with our methodological partners.

#### SR Conduct

The guideline practices survey revealed high standards for methodological rigor, consistency, and clarity among successful programs. To meet these standards, NCS will collaborate with a group of professional methodologists well versed in GRADE techniques. The society has committed to collaboration with Guidelines in Intensive Care, Development and Evaluation (GUIDE), a group of methodologists at McMaster University (Hamilton, Ontario, Canada). GUIDE will perform the entire SR and provide instruction in GRADE techniques to the team. Panelists will gain from the streamlined and shortened process and reduction in workload.

#### Consistent and Explicit Use of Evidence to Decision Framework

A defining feature and innovation of GRADE is a structured process for applying population-level evidence to the individual patient encounter, referred to as the Evidence to Decision Framework. This process is facilitated and made clear, deliberate, and explicit by the use of GRADE software, which is provided and managed by our external methodology partners.

#### Uniform Recommendation Format to Minimize Good Practice Statements

Successful guideline programs strive for a consistent and recognizable format and brand in their products, even if methodologies differ. This is particularly true for the presentation of recommendations. NCS will follow strict GRADE guidance in formulating recommendations and minimize the inclusion of good practice statements based on expert opinion.

### Conclusions

The GC performed a review of past NCS guideline practice and a survey of the practices of a number of other organizations. A principal finding was that most successful guideline programs incorporate professional methodologists into their working groups. By adopting this practice, NCS will be able to significantly improve the quality of its guidelines and establish a reliable, consistent, and trustworthy brand. To that end, the NCS Board has adopted the GC recommendation to collaborate with a team of external GRADE methodologists, an alliance that affirms the society’s commitment to developing high-quality resources to support and advance the practice of neurocritical care.

Embarking on this partnership, the GC anticipates three significant benefits. First, NCS members will find the experience to be more aligned with their expectations and hence will be more likely to volunteer their time on guideline development. Second, we expect guideline production to follow a more predictable and efficient timeline, with likely reduced time to publication. Last but not the least, our guidelines will achieve a consistent level of quality that can be favorably compared to those of other societies. This could greatly improve the dissemination and implementation of our guidelines.

## Supplementary Information

Below is the link to the electronic supplementary material.

Supplementary file1 (DOCX 59 kb)
