# Supplementary material for: Neurocritical Care Society Guidelines Update: Lessons from a Decade of GRADE Guidelines
Source: Neurocrit Care. 2021 Nov 2;36(1):1–10. doi: 10.1007/s12028-021-01375-1 (PMC8562933; doi:10.1007/s12028-021-01375-1)
Supplement: Supplementary file 1 — Supplementary file1 (DOCX 59 kb) [file 12028_2021_1375_MOESM1_ESM.docx]

# Supplemental Digital Content

## Table 1. GC Survey Topics and Questions (supplemental material)

| **Guidelines Committee** |
| --- |
| 1. Is there a specific committee for development/ generation of guidelines? |
| 1. How is committee organized? |
| 1. How are committee members selected? |
| 1. What is the committee budget? |
| 1. Any specific investment in process (such as methodological training [GRADE], tool license purchase [Distiller, EndNote, etc]) or otherwise? |
| 1. What is the committee structure? |
| 1. What is the committee purpose/tasks (guideline generation, review of endorsement requests, other)? |
| 1. Is there a specific committee for development/ generation of guidelines? If so, how is it organized? |
| **Guidelines** |
| 1. How are guideline topics identified? |
| 1. What is the timeline for projects? |
| 1. Do they have a routine interval for update of guidelines/documents? If so, what? If not, how do they approach this? |
| 1. How does the organization identify the type of document (guideline, consensus, white paper, or otherwise)? |
| 1. How does the organization handle collaborations (via MOU, joint-publication, # of writing committee members specified for the project, other)? |
| 1. How are writing committee members/participants identified/assigned? |
| 1. What support or training is offered for committee members? |
| 1. What is the committee member appointment/tenure duration? |
| 1. Is the systematic review portion of the process completed internally or externally (do committee members do this or is this commissioned to be done by an external entity)? |
| 1. Is a librarian used? |
| 1. Is there a standing contract between the librarian and the organization or are services arranged per project? |
| 1. What is the budget assigned per Guideline? |
| 1. Do they routinely perform meta-analyses? |
| 1. Do they report/display Forest plots? |
| 1. Who provides statistical support? |
| **Methodology** |
| 1. How does their org decide on scope (number of PICOs, repeated according to varying populations, or otherwise?) |
| 1. Does their org use GRADE? |
| 1. Does their org use GRADE rigorously or do they interpret more loosely? |
| 1. What is their process to make recommendations if not using GRADE? |
| **How do they address conflict resolution** |
| 1. during screening of literature? |
| 1. during recommendation generation? |
| **Format of Guideline** |
| 1. Is a Medical Writer/Editor utilized? |
| 1. How are Summary of Findings tables formatted? |
| 1. How are Risk of Bias tables formatted? |
| 1. How do they formulate recommendations (following GRADE or do they use some specific nomenclature for their recommendations – including format)? |
| 1. Do they distinguish recommendations from good practice statements? If so, how? |
| 1. Do they have a resource/manual for the guideline group and or writing committees? Can we get access to it if not published? |
| 1. How are their products marketed/disseminated? |
